# *BCL2* is a major regulator of haploidy maintenance in murine embryonic stem cells

**DOI:** 10.1111/cpr.13498

**Published:** 2023-05-05

**Authors:** Shengyi Sun, Qin Zhao, Yiding Zhao, Mengyang Geng, Qing Wang, Qian Gao, Xiao‐Ou Zhang, Wenhao Zhang, Ling Shuai

**Affiliations:** ^1^ State Key Laboratory of Medicinal Chemical Biology, College of Pharmacy, Tianjin Central Hospital of Gynecology Obstetrics/Tianjin Key Laboratory of Human Development and Reproductive Regulation Nankai University Tianjin China; ^2^ Shanghai Key Laboratory of Maternal and Fetal Medicine, Clinical and Translational Research Center of Shanghai First Maternity and Infant Hospital, Frontier Science Center for Stem Cell Research, School of Life and Science and Technology Tongji University Shanghai China; ^3^ Chongqing Key Laboratory of Human Embryo Engineering Chongqing Health Center for Women and Children Chongqing China; ^4^ National Clinical Research Center for Obstetrics and Gynecology Peking University Third Hospital Beijing China

## Abstract

Mammalian haploid cells are important resources for forward genetic screening and are important in genetic medicine and drug development. However, the self‐diploidization of murine haploid embryonic stem cells (haESCs) during daily culture or differentiation jeopardizes their use in genetic approaches. Here, we show that overexpression (OE) of an antiapoptosis gene, *BCL2*, in haESCs robustly ensures their haploidy maintenance in various situations, even under strict differentiation in vivo (embryonic 10.5 chimeric fetus or 21‐day teratoma). Haploid cell lines of many lineages, including epiblasts, trophectodermal lineages, and neuroectodermal lineages, can be easily derived by the differentiation of *BCL2*‐OE haESCs in vitro. Transcriptome analysis revealed that *BCL2*‐OE activates another regulatory gene, *Has2*, which is also sufficient for haploidy maintenance. Together, our findings provide an effective and secure strategy to reduce diploidization during differentiation, which will contribute to the generation of haploid cell lines of the desired lineage and related genetic screening.

## Graphical abstract

*BCL2*‐OE haESCs present reduced diploidization and sustain haploidy stably in differentiated cells, with an intact genome. A *BCL2*‐related gene, *Has2*, is also sufficient to haploidy maintenance. These findings provide a premium and secure strategy for haploidy maintenance of mouse haESCs, guaranteeing their advantages in genetic approaches.

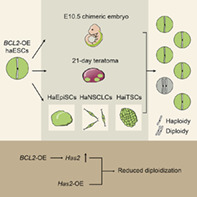

## 1 INTRODUCTION

In the past decade, haploid embryonic stem cells (haESCs) have been achieved in many species, including mice and humans^1^, ^2^; these cells contribute to advances in forward genetic screening due to their hemizygous stem cell properties.^3^, ^4^ However, haESCs tend to revert to diploid genomes in daily culture; thus, periodic and complicated fluorescence‐activated cell sorting for haploid enrichment is necessary. This is a time‐consuming and costly experimental strategy for many researchers using haESCs for desired genetic approaches. To date, many groups have attempted to elucidate the possible mechanisms of self‐diploidization, such as an abnormal cell cycle,^5^ prolonged mitosis,^6^ and different energy metabolism.^7^ To address these issues, researchers have used various methods to avoid the diploidization of haESCs, including adding chemical compounds to the medium^8^, ^9^ or editing critical genes.^10^, ^11^, ^12^ To the best of our knowledge, *p53* deletion is an efficient method to stabilize haploidy in cell cultures, facilitating the derivation of haploid cell lines in other cell types.^13^, ^14^ Nevertheless, *p53* is a tumour‐related gene that might result in an unstable genome.^15^ In addition, haESCs with *p53* disruption could not sustain haploidy under rigorous differentiation in vivo, such as in long‐term teratoma formation.^12^ Hence, whether there is another effective method to more efficiently prevent diploidization is an urgent issue.

Previous reports showed that *p53*‐null haESCs presented better cell viability than wild‐type (WT) haESCs in daily culture and differentiation^11^, ^12^; thus, it is interesting to address whether apoptosis genes are effective for maintaining haploidy. *BCL2* is a gene encoding a member of the B‐cell lymphoma 2 protein family that regulates cell death in multiple cell types with antiapoptotic roles.^16^ Overexpression (OE) of *BCL2* (*BCL2* OE) in mouse epiblast stem cells (EpiSCs) and human ESCs could dramatically improve cell viabilities and enable them to contribute to mouse chimeric embryos,^17^, ^18^ indicating that *BCL2* was an effective anti‐cell death regulator without affecting pluripotency. However, whether *BCL2* OE can improve the survival of haESCs during routine culture and differentiation to stabilize haploidy is still unclear.

In this study, we introduced exogenous *BCL2* into haESCs via the *piggyBac* (PB) system and compared the haploid maintenance of *BCL2*‐OE haESCs with that of WT haESCs in many aspects. Thereafter, we attempted to address the potential mechanism underlying how *BCL2*‐OE stabilized haploidy by RNA‐seq analysis and validation experiments.

## 2 MATERIALS AND METHODS

### 2.1 Mice

All specific pathogen‐free‐grade mice were purchased from Beijing Vital River Laboratory Animal Technology Co., Ltd., and used according to the Institutional Animal Care and Use Committee of Nankai University.

### 2.2 Cell culture and cell sorting

The mouse parthenogenetic haESCs and diploid ESCs were established in our laboratory previously with a serum‐modified medium.^19^ The ESCs were cultured on feeder cells and passaged every other day, with the medium changed every day. For the formation of embryoid bodies (EBs), ESCs were trypsinized to single cells and float cultured in serum medium without PD0325901 (MCE, HY‐10254), CHIR99021 (MCE, HY‐10102) and mouse LIF (SinoBiological, 50,756‐MNAH). The haEpiSC derivation and culture were described in our previous report.^13^ The culture medium for differentiated cells from teratomas was Ndiff. Medium (TaKaRa, Y40002) with 10 ng/mL EGF (PeproTech, 31509) and 10 ng/mL bFGF (PeproTech, 100‐18B). For a generation of haNSCLCs, *BCL2*‐OE haESCs were cultured with Ndiff. Medium (TaKaRa, Y40002) in non‐TC dishes (NEST, 706011) for aggregation. After 7 days, the EBs were plated in Matrigel (BD, 354230)‐precoated TC dishes (NEST, 706001) and further cultured in Ndiff. medium with 10 ng/mL EGF (PeproTech, AF‐315‐09‐100) and 10 ng/mL bFGF (PeproTech, AF‐100‐18B‐100) for expansion. The culture and respective medium of iTSCs were prepared according to a previous protocol.^20^

For enrichment of haploid cells, the cells were trypsinized into single cells, incubated with 4 μg/mL Hoechst 33342 (Thermo. H3570) at 37°C for 20 min, and sorted on a cell sorter (Beckman, MoFlo Astrios EQ).

For calculating the percentage of haploid cells, a previous report was referenced.^21^ Briefly, the 1n‐peak in haploid cells was the haploid cells with a 1‐copy DNA set at G1 phase (*a*%); the 2n‐peak in haploid cells was the haploid cells with a 2‐copy DNA set at S‐G2/M phase, mixing with diploid cells at G1 phase (*b*%); the 4n‐peak in haploid cells was the diploid cells with a 2‐copy DNA set at S‐G2/M phase (*c*%) (Figure S1F).

If *x*% was the percentage of diploid cells at the G1 phase in 2n‐peak, the percentage of haploid S‐G2/M cells was:

Haploid cells in (S‐G2/M) = *b*% − *x*%.

The percentage of total haploid cells was thus:

Total haploid cells = *a*% + (*b*% − *x*%).

In the diploidized cells, the 2n‐peak in diploid cells was the diploid cells with a 2‐copy DNA set at G1 phase (*d*%); the 4n‐peak in haploid cells was the diploid cells with a 2‐copy DNA set at S‐G2/M phase (*e*%) (Figure S1F).

We assumed that the cell division cycles of diploidized cells among haploid cells were the same as those of WT diploid cells. The ratio of *x*% to *c*% in haploid cells was equal to the ratio of *d*% to *e*% in diploidized cells: *x*%/*c*% = *d*%/*e*%.

So, *x*% was thus:

*x*% = *c*% * *d*%/*e*%.

The percentage of haploid cells was equal to

*a*% + (*b*% − *c*% * *d*%/*e*%).

### 2.3 Vector construction and electroporation

For the OE vectors, the coding sequence (CDS) of a specific gene was ligated to a modified *PiggyBac* vector (SBI, PB513B‐1) as indicated. The PBase plasmid (SBI, PB210PA‐1) was purchased from a local agent (Figure S1A). For KO plasmids, specific sgRNAs were designed according to the online software (http://crispor.tefor.net/crispor.py), whose oligos were phosphorylated with T4PNK (TaKaRa, 2021A), annealed, and ligated to linearized pSpCas9(BB)‐2A‐GFP (PX458) (Addgene, 48138). For electroporation, approximately 1 × 10^6^ cells were transfected with 6 μg plasmids on an electroporator (Thermo, Neon) at 1400 V, 10 ms, and 3 pulses.

### 2.4 Quantitative PCR, Western blotting, and immunostaining

For qPCR, total RNA from each sample was prepared using TRIzol reagent (Thermo, 317110), and cDNA was obtained using a Prime ScriptTM RT Reagent Kit with gDNA Eraser (TaKaRa, RR047A). For miRNA first‐strand cDNA synthesis, a tailing reaction kit was used (Sangon Biotech, B532451). qPCR was performed with Hieff™ SYBR Green Master Mix (Yeasen, 11201ES08). The mRNAs were normalized to *Gapdh* and miRNA were normalized to *U6*. Averages and SD values were based on repeats in triplicate.

For WB, protein samples from ESCs were extracted by RIPA lysis solution (Solarbio, R0010). Lysates were precleared by centrifugation for 5 min. Equal amounts of cell lysates were separated by SDS–PAGE and transferred to a polyvinylidene fluoride (PVDF) membrane (GE, 10600023). The membranes were blocked in 5% nonfat dry milk for 1 h, washed three times with TBST, and incubated with primary antibodies against BCL2 (Abclonal, A0208) and GAPDH (Abclonal, AC001) overnight at 4°C. Then, the membranes were washed three times and incubated with secondary antibodies for 2 h at room temperature (RT). The washed membranes were detected by a cECL Western Blot Kit (CWbiotech, CW0048M) and imaged.

For immunostaining, cells were fixed with 4% paraformaldehyde (Sigma, P6148) at RT for 1 h, permeabilized with 0.5% Triton X‐100 (Sigma, T8787) for 1 h, and blocked in 2% BSA for 2 h and incubated with primary antibodies against TFE3 (Sigma, HPA023881), PAX6 (Abcam, ab5790) and CDX2 (Abclonal, A1629) at 4°C overnight. After being washed three times, the cells were stained with secondary antibodies at RT for 1 h. After another three wash steps, the nuclei were stained with DAPI (Yeasen, 40727ES10) for 10 min at RT. Images were captured with a confocal microscope (Leica, SP8).

### 2.5 Chimeric assay and teratoma formation

E6.5, E8.5, and E10.5 chimeric embryos were generated by injecting GFP‐labelled *BCL2‐OE* haESCs or GFP‐labelled WT haESCs into CD‐1 background blastocysts. Reconstructed embryos were transferred to the oviducts of pseudopregnant mice at 0.5 dpc. At E6.5, E8.5, and E10.5, pseudopregnant mice were sacrificed to dissect chimeric embryos. Chimeric embryos were trypsinized into single cells, which were further analysed for GFP positivity and DNA content.

For teratoma analysis, approximately 1 × 10^7^ GFP‐labelled *BCL2*‐OE haESCs or *p53*‐KO haESCs or *Has2*‐OE haESCs or GFP‐labelled WT haESCs were injected subcutaneously into the limb of a 6‐week‐old male SCID mouse. Half of the fully formed teratoma was dissected 21 days later and stained with H&E for further analysis. Another half of the fully formed teratoma was dissected to analyse the DNA content.

### 2.6 Inverse PCR

Inverse PCR was used to identify the PB insertion sites. Genomic DNA extracted from cells was digested by PsuI (Thermo, FD1554). The fragments were incubated with T4 ligase (TaKaRa, 6023) at 16°C for 16 h and purified by a kit (Sangon Biotech, B518141). The inverse PCR conditions were as follows: 98°C for 2 min; 35 cycles at 98°C for 10 s, 55°C for 5 s, 72°C for 30 s, and finally 4°C. The products of the first round of PCR were used as the template for the second round of PCR under the same conditions. All the primers used in this research are summarized in Table S1.

### 2.7 Growth curve, haploidy analysis, cell viability, and caspase‐3 activity

For the cell survival assay, 1 × 10^4^ haploid cells were sorted and plated in a 24‐well plate. Each group was assayed three times. After 24 h, the cells were dissociated and counted with a cell counter system (Logos Biosystems, LUNAII). The number of living cells was recorded every other day to describe the cell growth curve. The total numbers of each sample were compared statistically. Sorted haESCs were passaged every 2–3 days to analyse the rate of diploidization. The percentages of haploid cells were analysed on Day 7, Day 14, and Day 21 according to DNA content and calculated based on a previous report.^21^ For cell viability analysis, cells were assessed in 24‐well plates (NEST, 702001) using Cell Counting Kit‐8 (Yeasen, 40203ES76). For caspase‐3 activity, 2 × 10^6^ cells were detected by the Caspase‐3 Activity Assay Kit (Beyotime, C1116) following the instructions. Averages and SD values were calculated based on three repeats.

### 2.8 Analysis of RNA‐seq and CNV data

All the RNA‐seq and CGH (or copy number vibration [CNV]) data were sequenced in a local company (Novogene). For RNA‐seq, clean data were used for subsequent analysis. Reference genome mm39 and gene model annotation files were downloaded from the UCSC and gene code websites directly. The index of the reference genome was built, and paired‐end clean reads were aligned to the reference genome using STAR software. HTseq was used to count and output the number of reads. For identification of the DEGs among different samples, normalized counts were calculated by the DESeq2 R package. The RNA‐seq data of WT diESCs, WT haESCs, and *p53*‐KO haESCs came from previous papers.^7^, ^12^, ^14^

CNV date identification was based on the GATK best practice guidelines as previously described.^22^ Briefly, paired‐end reads were aligned to the mm10 mouse reference genome with BWA mem, and duplicates were removed using Picard. The mouse reference genome was then segmented into 1 kb bins, and whole genome sequencing reads in each bin was summarized. With the male mouse kidney sample as the normal control, the binned read counts were denoised, and copy ratios were called for each sample. Contiguous segments were further merged, and copy ratios were reassigned with a Gaussian‐kernel binary segmentation algorithm.

## 3 RESULTS

### 3.1 BCL2 OE can enhance the cell survival abilities of haESCs

**FIGURE 1:**
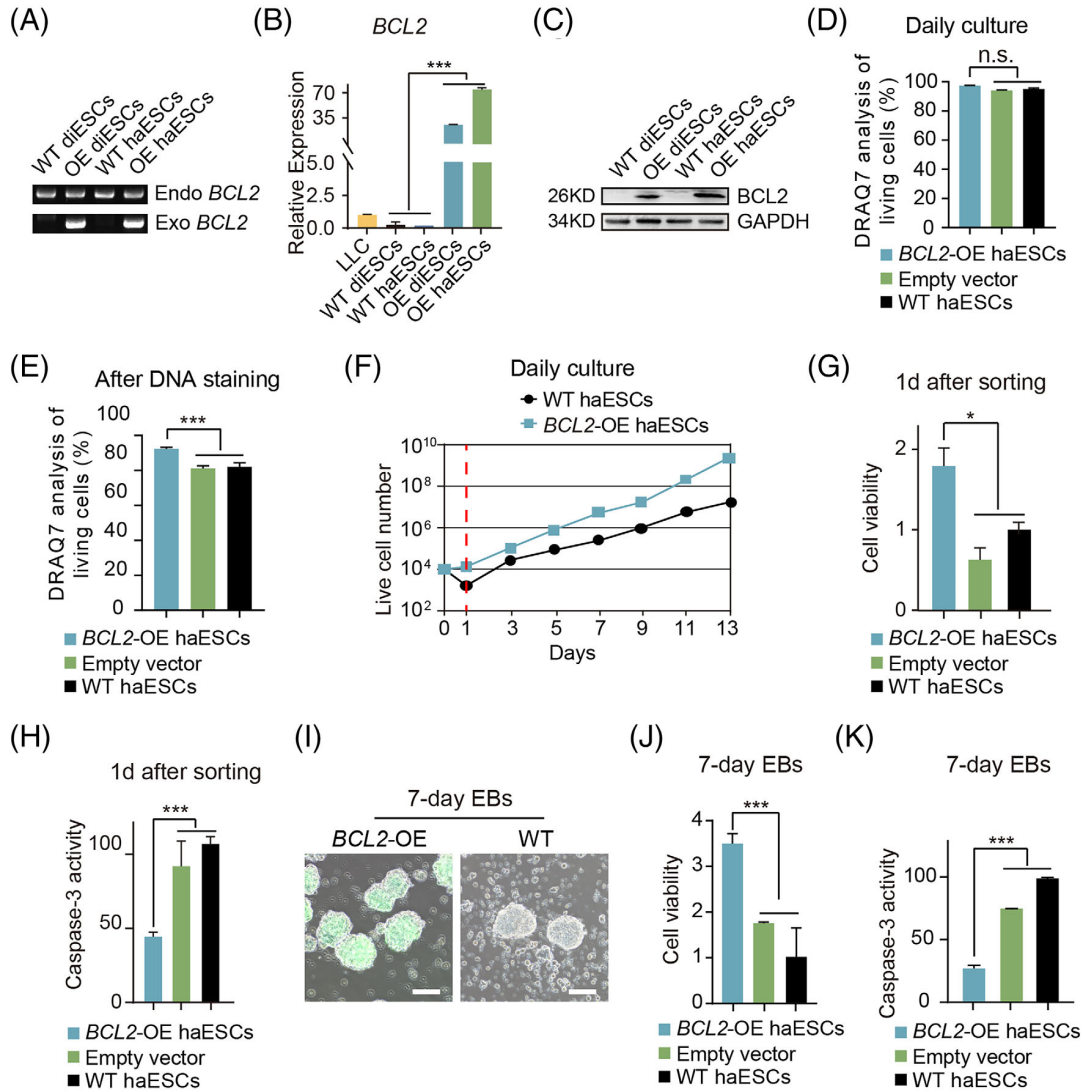
Improvement of the viability of *BCL2*‐OE HaESCs. (A) Genotyping identification of OE haESCs and OE diESCs. WT haESCs and WT diESCs were used as controls. (B) Expression levels of *BCL2* in LLC, WT diESCs, OE diESCs, WT haESCs, and OE haESCs cells determined by qPCR (*n* = 3 independent experiments). T‐test, ***p < 0.001. Data are represented as the mean ± SEM. (C) WB results of *BCL2* in WT diESCs, OE diESCs, WT haESCs, and OE haESCs cells. GAPDH was used as a loading control. (D) The percentage of living cells in *BCL2*‐OE haESCs in daily culture detected by DRAQ7 analysis, with PB‐GFP vector‐transfected haESCs and WT haESCs as controls (*n* = 3 independent experiments). T‐test, n.s., no significant difference. Data are presented as the mean ± SEM. (E) The percentage of living cells in *BCL2*‐OE haESCs after DNA staining detected by DRAQ7 analysis, with PB‐GFP vector‐transfected haESCs and WT haESCs as controls (*n* = 3 independent experiments). T‐test, ***p < 0.001. Data are presented as the mean ± SEM. (F) The live cell number of WT haESCs and *BCL2*‐OE haESCs in daily culture after sorting. *BCL2*‐OE haESCs survived more than WT haESCs 1 day after sorting but did not show an obvious expansion advantage over WT haESCs later. (G) The cell viability of *BCL2*‐OE haESCs and PB‐GFP vector‐transfected haESCs 1 day after sorting detected by CCK8 assays, with WT haESCs as a control. (*n* = 3 independent experiments). T‐test, *p < 0.05, n.s., no significant difference. Data are represented as the mean ± SEM. (H) Caspase‐3 activity of *BCL2*‐OE haESCs and PB‐GFP vector‐transfected haESCs 1 day after sorting, with WT haESCs as a control (*n* = 3 independent experiments). T‐test, ***p < 0.001. Data are presented as the mean ± SEM. (I) Image of haEBs (bright field and GFP positive) derived from *BCL2*‐OE haESCs and image of haEBs derived from WT haESCs after 7 days. Scale bar, 100 μm. (J) The cell viability of *BCL2*‐OE haEBs and PB‐GFP vector‐transfected haEBs on Day 7 detected by CCK8 assays, with WT haEBs as a control. T‐test, **p < 0.01, n.s., no significant difference. Data are presented as the mean ± SEM. (K) Caspase‐3 activity of *BCL2*‐OE haEBs and PB‐GFP vector‐transfected haEBs on Day 7, with WT haEBs as a control (*n* = 3 independent experiments). T‐test: ***p < 0.001, n.s., no significant difference. Data are presented as the mean ± SEM.

To assess whether *BCL2* OE could improve the survival ability of haESCs under some harsh experimental conditions, we introduced exogenous *BCL2* into WT haESCs. Given that the PB transposon is efficient and widely used in delivering exogenous genes into the genome,^23^, ^24^ we constructed a *BCL2*‐OE vehicle based on a PB vector carrying a *GFP* gene (Figure S1A). Thereafter, the *BCL2*‐OE plasmids were electroporated into mouse WT haESCs established in our laboratory previously.^19^ Three days after electroporation, 9.9% of GFP‐positive cells were enriched by FACS (Figure S1B). The sorted cells were expanded and stably showed green fluorescence during long‐term culture (Figures S1C and S1D). We also introduced the *BCL2*‐OE plasmids into a diploid ESC line (diESCs) with the same protocol to detect exogenous *BCL2* by genomic PCR (Figure 1A). The quantitative PCR (qPCR) results demonstrated that the expression levels of *BCL2* in both *BCL2*‐OE haESCs and *BCL2*‐OE diESCs were much higher than those in WT ESCs and a mouse lung tumour line (LLC, highly expressing *BCL2*) (Figure 1B). The Western blotting (WB) results further confirmed this result (Figure 1C). Although *BCL2*‐OE haESCs did not show an obvious viability advantage compared with WT haESCs in daily culture and passage (Figures 1D and S1E), we attempted to address whether *BCL2*‐OE haESCs presented higher survival abilities in harsh conditions. The DNA content‐based FACS method for haploid enrichment was harmful to cells (the DNA staining buffer Hoechst 33342 was cytotoxic), which resulted in severe cell death after staining and subsequent sorting.^25^ DRAQ7 analysis showed that *BCL2*‐OE haESCs presented obviously reduced apoptosis compared to WT haESCs after DNA staining (Figure 1E). Thereafter, we performed FACS for haploid enrichment and sorted equal cell amounts of *BCL2*‐OE and WT haESCs (10,000 cells per sample) to plate back for further culture. Interestingly, the surviving cells of *BCL2*‐OE haESCs were much more than those of WT haESCs 1 day after sorting (Figure 1F). Next, we detected the cell viability and apoptosis of the cells 1 day after sorting by CCK8 and caspase‐3 activity analysis and found that *BCL2*‐OE haESCs indeed presented better viability and reduced apoptosis compared with WT haESCs (Figure 1G, H). To address whether *BCL2*‐OE haESCs showed better survival than WT haESCs during random differentiation in vitro, we performed embryoid body (EB) aggregation assays with these cells. Although the 7‐day EBs from *BCL2*‐OE haESCs and WT haESCs did not show obvious differences in morphology (Figure 1I), the *BCL2*‐OE haESCs presented better survival in CCK8 and caspase‐3 analysis (Figure 1J, K). All the results indicated that the antiapoptotic ability of *BCL2*‐OE haESCs was drastically improved under harsh conditions.

### 3.2 BCL2‐OE haESCs maintain stable haploidy during daily culture

**FIGURE 2:**
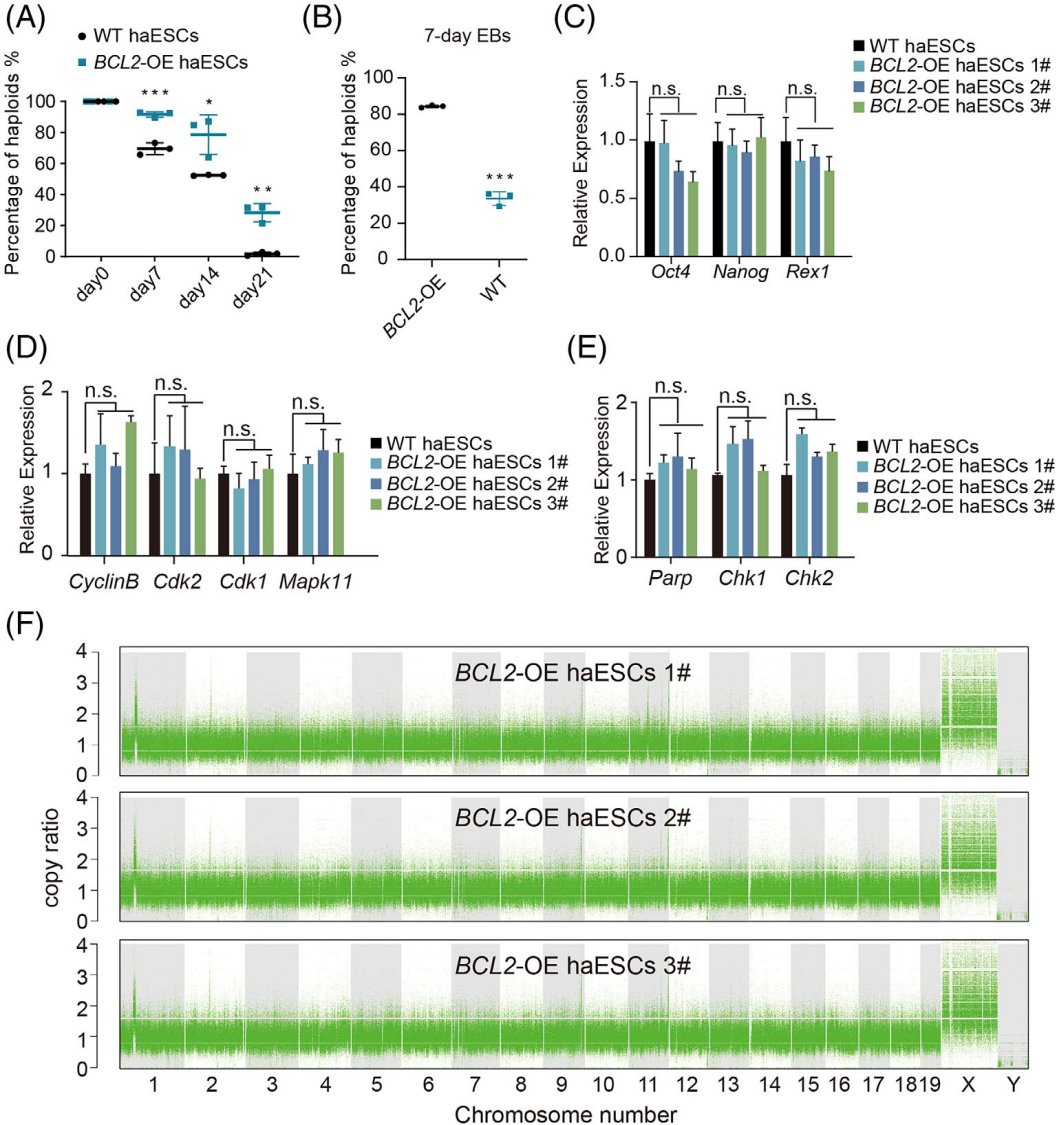
Characteristics of *BCL2*‐OE HaESCs. (A) The percentage of haploid cells in WT haESCs and *BCL2*‐OE haESC lines during daily culture. T‐test, *p < 0.05, **p < 0.01, ***p < 0.001. Data are represented as the mean ± SEM. (B) The percentage of haploid cells in *BCL2*‐OE haEBs and WT haEBs on Day 7. T‐test, ***p < 0.001. Data are presented as the mean ± SEM. (C) Expression levels of pluripotent genes (*Oct4*, *Nanog*, and *Rex1*) in WT haESCs and *BCL2*‐OE haESCs determined by qPCR. T‐test, n.s., no significant difference. Data are represented as the mean ± SEM. (D) Expression levels of cell cycle‐related genes (*Cyclin B*, *Cdk1*, *Cdk2*, and *Mapk11*) in WT haESCs and three corresponding *BCL2*‐OE haESC lines determined by qPCR. T‐test, n.s., no significant difference. Data are represented as the mean ± SEM. (E) Expression levels of DNA damage‐related genes (*Parp*, *Chk1*, and *Chk2*) in WT haESCs and *BCL2*‐OE haESC lines determined by qPCR. T‐test, n.s., no significant difference. Data are represented as the mean ± SEM. (F) Copy number vibration analysis of *BCL2*‐OE haESCs. The 129Sv/Jae male mouse kidney was used as a control.

To address haploidy maintenance, we randomly selected three subclones of *BCL2*‐OE haESCs and tested whether they exhibited stability in haploid maintenance during daily culture without sorting for 3 weeks compared with WT haESCs. The FACS analysis results in every 7 days showed that the proportion of haploid cells in *BCL2*‐OE haESCs was much higher than that in WT haESCs (Figure 2A), demonstrating that *BCL2*‐OE was able to enhance haploid maintenance during long‐term culture. Thereafter, we analysed the haploid maintenance of EB aggregates in a 7‐day random differentiation. The results demonstrated that the *BCL2*‐OE group also had advantages in haploid maintenance during random in vitro compared with WT haESCs (Figure 2B). Next, whether *BCL2* OE affected the expression of some key genes in haESCs was measured. The qPCR results showed that there was no significant difference in the expression levels of pluripotency‐, cell cycle‐ and DNA damage‐specific genes between *BCL2*‐OE haESCs and WT haESCs (Figure 2C, D, E).

To further assess the genomic integrity of *BCL2*‐OE haESCs, we compared them with a male mouse kidney from the 129Sv/Jae strain using deep sequencing and CNV analysis. The results revealed that our *BCL2*‐OE haESCs did not present obvious copy number duplication or deletion in a haploid state (Figure 2F), indicating that *BCL2* OE maintained genomic integrity in cell culture without genomic mutations (Figure 2F). Thereafter, the insertion sites of PB in the three *BCL2‐*OE cell lines (1#, 2#, and 3#) were detected by inverse PCR, and the results showed that all the insertion sites were located in noncoding regions or nonharmful regions (Figures S2A and S2B). Thus, *BCL2* OE can significantly improve the haploid maintenance during daily culture and random differentiation in vitro without any obvious mutation.

### 3.3 BCL2‐OE haESCs stably maintain haploidy during differentiation in vivo and in vitro

**FIGURE 3:**
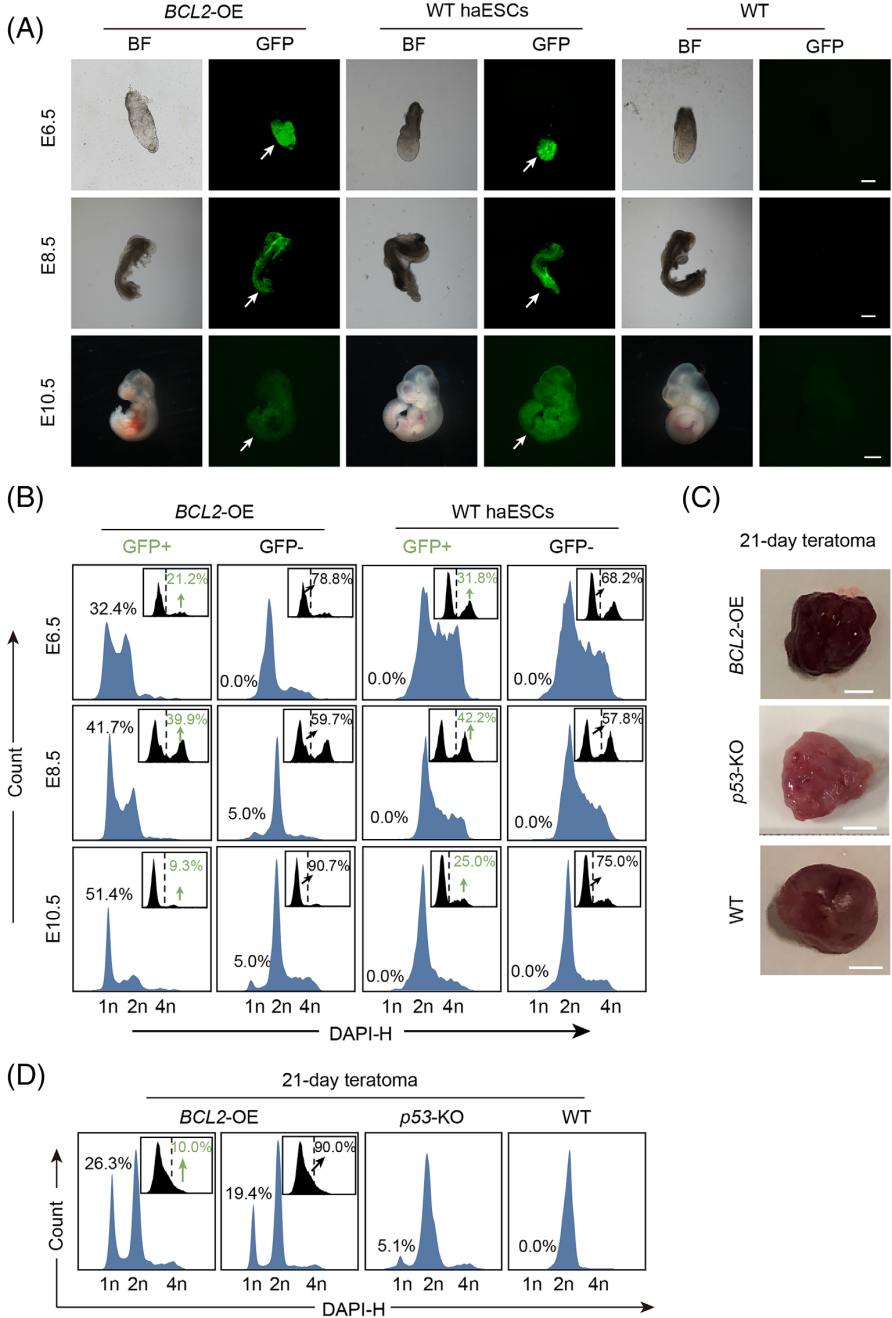
Haploid stability of *BCL2*‐OE HaESCs in vivo. (A) Morphology of chimeric embryos at E6.5, E8.5, and E10.5 derived from GFP‐labelled *BCL2*‐OE haESCs and WT haESCs. Scale bar, E6.5 and E8.5, 100 μm; E10.5, 1 mm. WT embryos were used as controls. (B) DNA content analysis of GFP+ cells and GFP‐ cells in chimeric E10.5 embryos derived from *BCL2*‐OE haESCs and WT haESCs. The GFP+ cells from chimeric embryos maintained a high percentage of haploid cells at the 1n peak (G0/G1 phase) in the *BCL2*‐OE haESC group. WT haESCs with GFP‐labelling were used as controls. (C) Teratomas formed from *BCL2*‐OE haESCs, *p53*‐KO haESCs and WT haESCs on Day 21. Scale bar, 1 cm. (D) DNA content analysis of GFP+ cells and GFP‐ cells in 21‐day teratomas derived from *BCL2*‐OE haESCs, *p53*‐KO haESCs and WT haESCs. The GFP+ cells from the *BCL2*‐OE teratoma maintained a high percentage of haploid cells at the 1n peak (G0/G1 phase).

We further investigated whether *BCL2* OE could ensure haploid maintenance during differentiation in vivo. We microinjected GFP‐labelled *BCL2‐OE* haESCs into blastocysts to construct chimeric embryos, with GFP‐labelled WT haESCs as controls. The reconstructed embryos were transferred into the oviducts of 0.5‐day postcoitum (dpc) pseudopregnant mice. At embryonic Days 6.5, 8.5, and 10.5 (E6.5, E8.5, and E10.5), pseudopregnant mice were sacrificed to analyse the chimeric embryos. The chimeric embryos derived from *BCL2*‐OE haESCs and WT haESCs were similar to WT embryos at E6.5, E8.5, and E10.5 morphologically with the contribution of GFP‐positive cells (Figure 3A, B). The FACS results showed that 21.2%, 39.9%, and 9.3% of GFP‐positive cells existed in the E6.5, 8.5, and 10.5 *BCL2‐OE* haESC chimeric embryos, and 31.8%, 42.2%, and 25.0% of GFP‐positive cells existed in the E6.5, 8.5 and 10.5 WT haESC chimeric embryos, respectively. According to the DNA content analysis, there was still a high proportion of haploid cells (1n peak) in the E6.5, 8.5, and 10.5 GFP‐positive cells from *BCL2‐OE* haESC chimeric embryos (Figure 3B), while there were no haploid cells in those from WT haESC chimeric embryos. This result indicated that *BCL2*‐OE haESCs could further differentiate into a haploid genome with embryonic development potential. We also analysed GFP‐negative cells in *BCL2‐OE* haESC chimeric embryos and found that there was also a small population of haploid cells among them, probably gradually silencing GFP‐positive cells (Figure 3B).

To assess the pluripotency of *BCL2‐*OE haESCs to three germ layers, we subcutaneously injected approximately 1 × 10^7^ cells into the limb of a 6‐week‐old male SCID mouse to form teratomas. Twenty‐one days later, normal teratomas were formed, similar to those from *p53*‐KO haESCs and WT haESCs (Figure 3C). The histological analysis results showed that there were glands (endoderm), muscles (mesoderm), and neural tube tissues (ectoderm) in the teratoma from *BCL2‐*OE haESCs (Figure S3A), suggesting that *BCL2‐*OE haESCs had the potential to differentiate into three germ layers. Next, we assessed the DNA contents of *BCL2‐*OE haESC‐derived teratomas by FACS analysis, with *p53*‐KO haESC‐derived teratomas as a control. Although there were no haploid cells in the WT teratoma and 5.1% of cells in the *p53*‐KO teratoma at the 1n peak, the percentage of the 1n peak in GFP‐positive cells from the *BCL2*‐OE teratoma was up to 26.3%. Interestingly, 19.4% of the GFP‐negative cells from the *BCL2*‐OE teratoma were also at the 1n peak (Figure 3D). To determine whether the GFP‐negative cells from *BCL2*‐OE teratomas were PB silenced or lost, we performed genomic PCR and qPCR to address this issue. Genomic PCR showed that the PB was still in the GFP‐negative cells from the *BCL2*‐OE teratoma, whereas they no longer expressed *BCL2* and *GFP* (Figure S3B and S3C). This result indicated that the *BCL2*‐OE vectors were silenced gradually during differentiation, which might be the reason that the GFP‐negative cells still had a small group of haploid cells during differentiation. Nevertheless, all the above results showed the great potential of *BCL2‐*OE haESCs in sustaining haploidy during long‐term differentiation.

**FIGURE 4:**
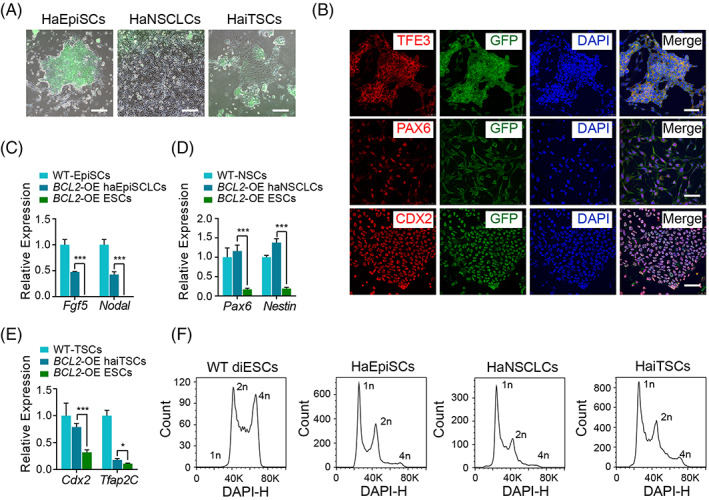
Haploid stability of *BCL2*‐OE HaESCs in vitro. (A) Bright field and GFP merged images of haEpiSCs, haNSCLCs and haiTSCs derived from *BCL2*‐OE haESCs. Scale bar, 100 μm. (B) Immunofluorescence of TFE3 in haEpiSCs, PAX6 in haNSCLCs and CDX2 in haiTSCs. DNA was stained with DAPI. Scale bar, 50 μm. (C)–(E) Expression levels of EpiSC‐specific genes (*Fgf5* and *Nodal*) in WT‐EpiSCs, *BCL2*‐OE haEpiSCs and *BCL2*‐OE haESCs; NSC‐specific genes (*Pax6* and *Nestin*) in WT‐NSCs, *BCL2*‐OE haNSCLCs and *BCL2*‐OE haESCs; TE‐specific genes (*Cdx2* and *Tfap2C*) in WT‐TSCs, *BCL2*‐OE haiTSCs and *BCL2*‐OE haESCs. Data are presented as the mean ± SEM. T‐test: *p < 0.05, ***p < 0.001. (F) DNA content analysis of haEpiSCs, haNSCLCs and haiTSCs derived from *BCL2*‐OE haESCs. There were 21.7%, 25.2%, and 24.3% of cells at the 1n peak in haEpiSCs, haNSCLCs and haiTSCs, respectively.

To examine the feasibility of using *BCL2‐*OE haESCs to generate haploid cell lines of other lineages by differentiation in vitro, we performed specific differentiation of *BCL2‐*OE haESCs into epiblasts, trophectodermal lineages, and neuroectodermal lineages. Typical EpiSCs, neural stem cell‐like cells (NSCLCs), and induced trophoblast stem cells (iTSCs) (induced assisted with *Cdx2*‐overexpresssion^20^) could be derived from the *BCL2‐*OE haESCs by differentiation and presented good status morphologically (Figure 4A). Next, we assessed the lineage‐specific markers in haploid EpiSCs (haEpiSCs), haploid NSCLCs (haNSCLCs), and haploid iTSCs (haiTSCs) by immunostaining. The results showed that TFE3 in haEpiSCs was located in the cytoplasm rather than in the nucleus, indicating a primed pluripotent state. Positivity for PAX6 was observed in haNSCLCs and CDX2 was observed in haiTSCs, demonstrating their NSC and TSC properties, respectively (Figure 4B). The qPCR results also confirmed the molecular identities of these differentiated cells (Figure 4C, E). In another parallel experiment, we independently analysed the DNA contents of haEpiSCs, haNSCLCs, and haiTSCs. There were 21.7%, 25.2%, and 24.3% of cells at the 1n peak in haEpiSCs, haNSCLCs, and haiTSCs, respectively, suggesting that these derived haploid cell lines from *BCL2‐*OE haESCs could also maintain a haploid genome in vitro (Figure 4F).

### 3.4 Transcriptome analysis reveals that Has2 is a key gene involved in the haploidy maintenance of BCL2‐haESCs

**FIGURE 5:**
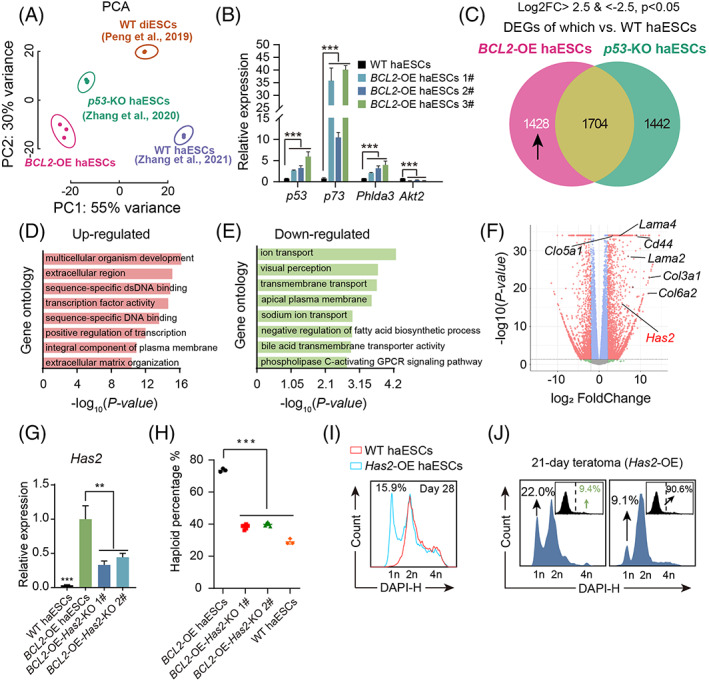
Transcriptome analysis of *BCL2*‐OE HaESCs. (A) PCA of *BCL2‐*OE haESCs, *p53‐*KO haESCs, WT haESCs and WT diESCs. The three *BCL2‐*OE haESC lines showed high similarity but were different from *p53‐*KO haESCs, WT haESCs and WT diESCs. (B) Expression levels of Akt‐p53 pathway‐related genes (*p53*, *p73*, *Phlda3*, and *Akt2*) in *BCL2‐*OE haESCs and WT haESCs determined by qPCR. T‐test, n.s., no significant difference, ***p < 0.001. Data are represented as the mean ± SEM. (C) The Venn diagram shows the DEGs between *BCL2‐*OE haESCs and *p53*‐KO haESCs versus WT haESCs. There were 1704 overlapping genes between *BCL2‐*OE haESCs and *p53*‐KO haESCs versus WT haESCs and 1428 *BCL2‐*OE haESCs only DEGs. (D) Gene ontology of the upregulated genes in 1428 *BCL2‐*OE haESC only DEGs. (E) Gene ontology of the downregulated genes in 1428 *BCL2‐*OE haESC only DEGs. (F) Volcano plot shows some extracellular matrix (ECM) related genes DEGs between *BCL2‐*OE haESC and WT haESCs. *BCL2* and *Has2* were in 1428 *BCL2‐*OE haESC only DEGs. (G) Expression levels of *Has2* in WT haESCs, *BCL2‐*OE haESCs and *BCL2‐*OE*‐Has2‐*KO haESCs. T‐test, **p < 0.01, ***p < 0.001. Data are represented as the mean ± SEM. *Has2* was significantly upregulated in *BCL2‐*OE haESCs and downregulated in *BCL2‐*OE*‐Has2‐*KO haESCs. (H) The percentages of haploid cells among WT haESCs, *BCL2‐*OE haESCs and *BCL2‐*OE*‐Has2‐*KO haESCs 7 days after sorting. The deletion of *Has2* impaired the stability of haploid maintenance in *BCL2*‐OE haESCs. (I) DNA content analysis of *Has2*‐OE haESCs and WT haESCs 28 days after sorting. (J) DNA content analysis of GFP+ cells and GFP‐ cells in 21‐day teratoma derived from *Has2*‐OE haESCs. The GFP+ cells from the teratoma maintained a high percentage of haploid cells at the 1n peak (G0/G1 phase).

To determine why *BCL2* OE can reduce the diploidization of haESCs, we analysed the transcriptome differences among *BCL2‐*OE, *p53*‐KO, and WT haESCs by RNA‐seq. The principal component analysis based on these samples showed that *BCL2‐*OE haESCs in three repeats were well clustered and were separated from *p53*‐KO haESCs,^12^ WT haESCs^7^ and WT diESCs^14^ (Figure 5A), suggesting that the relied‐on pathway for the stability of haploidy in *BCL2*‐haESCs might be different from that in *p53*‐KO haESCs. In addition, the qPCR results showed that the expression levels of *p53* and *p73* in *BCL2‐*OE haESCs were higher than those in WT haESCs, indicating that the haploid maintenance capability of *BCL2‐*OE haESCs was unrelated to *p53*‐KO (Figure 5B). The heatmap results showed that some extracellular matrix‐ (ECM‐) and cytoplasm‐related genes were upregulated in *BCL2‐*OE haESCs (Figure S4A). To deeply analyse the transcriptome of *BCL2‐*OE haESCs, we compared the differentially expressed genes (DEGs) between *BCL2‐*OE versus WT haESCs to the DEGs between *p53*‐KO haESCs versus WT haESCs. There were 1704 overlapping DEGs between *BCL2‐*OE and *p53*‐KO haESCs versus WT haESCs; however, we mainly focused on the remaining DEGs of *BCL2‐*OE haESCs versus WT haESCs (1428 DEGs) (Figure 5C). According to gene ontology analysis of the 1428 DEGs, the upregulated DEGs were mainly related to the extracellular region, ECM organization, and regulation of transcription, while the downregulated DEGs were enriched in the cellular transport process and other functions (Figure 5D, E).

To address the key genes involved in the haploid maintenance of *BCL2‐*OE haESCs, we focused on the ECM‐related genes among the DEGs of *BCL2*‐OE haESCs versus WT haESCs (Figure 5F). We found an interesting ECM‐related gene, *Has2*, being not involved in the DEGs between *p53*‐KO haESCs versus WT haESCs. *Has2* can encode hyaluronan synthase enzymes, which can synthesize an ECM molecule.^26^ To the best of our knowledge, *Has2* has not been reported to be related to the diploidization of haESCs. To further study the role of *Has2* in *BCL2‐*OE haESCs, we conducted *Has2* KO in *BCL2‐*OE haESCs via the *Has2*‐sgRNA CRISPR/Cas9 system and obtained *BCL2‐*OE‐*Has2*‐KO haESCs (Figures S4B and 5G). Interestingly, *BCL2‐*OE‐*Has2*‐KO haESCs showed a reduced haploid‐maintaining ability at the WT haESC level compared to *BCL2*‐OE haESCs (Figure 5H). It was important to determine whether *Has2* OE in WT haESCs itself could enhance the haploid maintenance ability. Thereafter, we introduced *Has2*‐OE vectors (based on a *PB* vector carrying the GFP gene) (Figure S4C) into WT haESCs by electroporation. The GFP+ cells showed higher *Has2* expression levels than WT haESCs, as shown by qPCR (Figure S4D). The DNA content analysis results demonstrated that compared with WT haESCs, *Has2‐*OE haESCs exhibited a very low rate of diploidization during long‐term (28‐day) culture (Figure 5I). In addition, *Has2‐*OE haESCs stably maintained haploidy in 21‐day teratomas, similar to *BCL2*‐OE haESCs (Figures S4E and 5J). Taken together, these results indicate that *Has2* itself is an essential gene regulating haploidy maintenance.

### 3.5 Comprehensive analysis shows that Has2‐OE inhibits apoptosis of HaESCs to prevent diploidization

**FIGURE 6:**
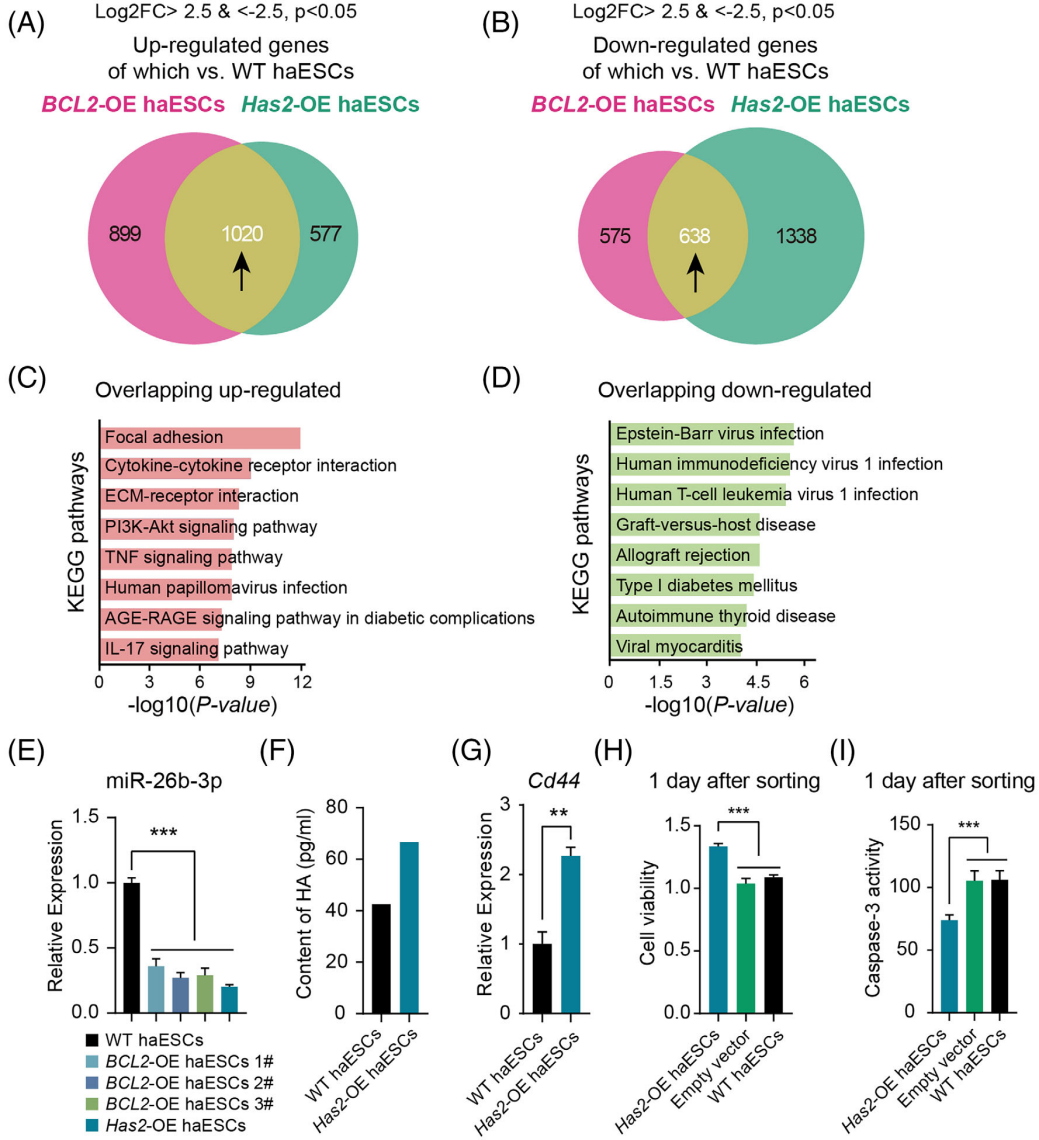
Comprehensive analysis of *Has2*‐OE HaESCs. (A) The Venn diagram shows the upregulated DEGs between *BCL2*‐OE haESCs and *Has2*‐OE haESCs versus WT haESCs. There were 1020 overlapping genes. (B) The Venn diagram shows the downregulated DEGs between *BCL2*‐OE haESCs and *Has2*‐OE haESCs versus WT haESCs. There were 638 overlapping genes. (C) KEGG analysis of upregulated genes in both *BCL2*‐OE and *Has2*‐OE haESCs. (D) KEGG analysis of downregulated genes in both *BCL2*‐OE and *Has2*‐OE haESCs. (E) Expression levels of miR‐26b‐3p in WT haESCs, *BCL2‐*OE haESCs and *Has2‐OE* haESCs. T‐test, ***p < 0.001. Data are represented as the mean ± SEM. (F) Content of HA in the medium of WT haESCs and *Has2‐OE* haESCs. (G) Expression levels of *Cd44* in WT haESCs and *Has2‐OE* haESCs. T‐test, **p < 0.01. Data are represented as the mean ± SEM. (H) The cell viability of *Has2*‐OE haESCs and PB‐GFP vector‐transfected haESCs 1 day after sorting detected by CCK8 assays, with WT haESCs as a control. (*n* = 3 independent experiments). T‐test, ***p < 0.001. Data are represented as the mean ± SEM. (I) Caspase‐3 activity of *Has2*‐OE haESCs and PB‐GFP vector‐transfected haESCs 1 day after sorting, with WT haESCs as a control (*n* = 3 independent experiments). T‐test, ***p < 0.001. Data are presented as the mean ± SEM.

According to the RNA‐seq data, *BCL2*‐OE haESCs had a high expression level of *Has2*, whereas *Has2*‐OE haESCs also had a high expression level of *BCL2* (Figure S5A). There was no significant difference in the expression levels of cell death‐related, pluripotent, cell cycle, and DNA damage genes between *Has2*‐OE haESCs and WT haESCs (Figure S5B, E). To further determine the reason that *Has2* OE and *BCL2* OE promote haploid maintenance in haESCs, we compared the DEGs between *Has2‐*OE haESCs versus WT haESCs to the DEGs between *BCL2*‐OE haESCs versus WT haESCs. There were 1020 overlapping upregulated DEGs and 638 downregulated overlapping DEGs between *BCL2*‐OE and *Has2*‐OE versus WT haESCs (Figure 6A, B). These upregulated overlapping DEGs were mainly related to ECM‐receptor interaction, focal adhesion, and the PI3K‐Akt signalling pathway, while the downregulated overlapping DEGs were enriched in Epstein–Barr virus infection and other functions according to KEGG analysis (Figure 6C, D). The results of gene set enrichment analysis demonstrated that the DEGs identified in *Has2*‐OE haESCs were strongly associated with ECM structural constituents, collagen‐containing ECM, and external encapsulating structure‐related genes (Figure S6A), which were also related to the ECM. These results were consistent with the results of the above quantitative analysis. The DEGs identified in *BCL2*‐OE haESCs were strongly associated with collagen‐containing ECM and collagen fibril organization (Figure S6B), which were also related to the ECM. However, whether ECM was a key reason for the stable sustaining of haploidy in *BCL2*‐OE haESCs and *Has2*‐OE haESCs warrants more investigation, which would be a research direction in the future.

Nevertheless, there was a possible explanation for the stable haploid maintenance of *Has2*‐OE haESCs. A previous report showed that miR‐26b enhanced ovarian granulosa cell apoptosis through the HAS2‐HA‐CD44‐Caspase‐3 pathway by targeting HAS2,^27^ and whether *Has2* OE could inhibit cell death of haESCs as *BCL2* OE was quite interesting. To investigate this hypothesis, we detected the expression levels of miR‐26b‐3p in *BCL2*‐OE, *Has2*‐OE haESCs, and WT haESCs. The results showed that both *BCL2* OE and *Has2* OE led to a reduction in miR‐26b expression (Figure 6E). We also found an increase in HA content in the medium of *Has2*‐OE haESCs and the upregulation of *Cd44* expression in *Has2*‐OE haESCs (Figure 6F, G). To confirm whether *Has2* OE resulted in antiapoptotic effects, we analysed cell viability 1 day after sorting using the same experiments as those for *BCL2* OE described above. The results of CCK8 and caspase‐3 activity analysis indicated that *Has2*‐OE haESCs also presented better viability and reduced apoptosis than WT haESCs (Figure 6H, I). Our results demonstrated that the potential mechanism underlying stable haploidy maintenance in *BCL2*‐OE haESCs might be antiapoptotic effects under harsh conditions through the HAS2‐HA‐CD44‐Caspase‐3 pathway by upregulating HAS2. Whether miR‐26b itself was critical for regulating haploidy maintenance was also very interesting, which would be studied in the future.

## 4 DISCUSSION

Approximately 20‐years‐ago, near‐haploid and modified haploid tumour cells began to be widely used in drug target or genetic screening, mainly for their single chromosome set, to easily produce genome‐wide hemizygous mutants.^28^, ^29^, ^30^, ^31^ Unlike haploid tumour cells, haESCs do not have genomic variations and have the potential to differentiate into any desired lineage^25^; thus, haESCs are promising in many more areas when used in genetic screening. However, frequent self‐diploidization of haESCs hinders their use in genetic approaches, which is why many people seek efficient methods to prevent diploidization of haESCs in culture.^5^, ^9^, ^32^ Nevertheless, previous methods to inhibit diploidization are either low‐efficiency or unsafe to the genome; therefore, an effective and secure strategy for haploid maintenance is needed. In our study, *BCL2*‐OE and *Has2*‐OE haESCs maintained haploidy without sorting for at least 3 weeks (Figures 2A and 5I). Notably, *BCL2*‐OE haESCs did not present genomic instability with any mutation by CNV analysis (Figure 2F), guaranteeing their value in developmental genetic screening. In addition, *BCL2*‐OE haESCs can contribute to normal chimeric embryos at E10.5 and differentiate into a 21‐day teratoma with a very high proportion of haploid cells (Figure 3). To the best of our knowledge, this is the longest term of differentiation in a haploid genome, contributing to tissues in vivo. For the failure of *BCL2*‐OE haESCs to contribute to further chimeric embryos (over E10.5) in subsequent development, it may be that the haploids themselves cannot form individuals. Another possible explanation for this is the silencing of exogenous *BCL2* with PB in the genome, eliminating the advantages of *BCL2*‐OE. This hypothesis is consistent with previous reports that transposons in the genome are always silenced during differentiation.^33^ Although we changed the promoter of *BCL2* in PB from EF1α to CAG, this issue has not yet been solved (data not shown). Other strategies including lentivirus or CRISPRa system would address this problem or not need further investigations.

Haploid cell lines of other lineages are powerful tools for lineage‐specific genetic screening. In our study, *BCL2*‐OE haESCs easily differentiated into haEpiSCs, haNSCLCs, and haiTSCs and maintained haploidy in further culture (Figure 4), which would be useful in specific genetic screening. Whether this system was of benefit for deriving haploid cell lines of other lineages requires more evidence. Regarding the mechanism underlying the stable maintenance of haploidy in *BCL2*‐OE and *Has2*‐OE haESCs, the expression of *p53* and *p73* was upregulated compared to that in WT haESCs, suggesting that they relied on completely different pathways for haploid maintenance from *p53*‐KO haESCs (Figures 5B and S6C). Thereafter, we performed *Has2* KO in *BCL2*‐OE haESCs and found that the expression levels of *p53* and *p73* in *BCL2*‐OE‐*Has2*‐KO haESCs were similar to those in WT haESCs, suggesting that the effect of *BCL2* on the upregulation of *p73* and *p53* was dependent on *Has2* (Figure S6D). Both *BCL2*‐OE haESCs and *Has2*‐OE haESCs enriched many upregulated genes in the ECM; thus, haploid maintenance might be related to cell adhesion or intercellular force. We attempted to culture WT haESCs in a feeder‐free system on Matrigel, fibronectin, gelatin, hyaluronic acid, or collagen‐precoated dishes. However, none of them had a validated positive correlation with haploid maintenance (data not shown). However, the downregulation of miR26b in *BCL2*‐OE haESCs and upregulation of HA and CD44 in *Has2*‐OE haESCs demonstrated that the activation of the HAS2‐HA‐CD44‐Caspase‐3 pathway might be the potential mechanism of stable maintenance of haploidy in *BCL2*‐OE and *Has2*‐OE haESCs.

## 5 CONCLUSION

In summary, our results show that *BCL2*‐OE haESCs could differentiate in an intact haploid genome by many assays both in vivo and in vitro. One of the most important reasons for stable haploid maintenance in *BCL2*‐OE haESCs is the upregulation of *Has2*, which itself is sufficient for haploid stability. Our findings safeguard the advantages of haESCs and facilitate the application of haESCs in many more specific genetic screens. However, genetic screening related to cell death using such a haploid system need more attention due to the anti‐cell death functions of *BCL2*‐OE and *Has2*‐OE.

## AUTHOR CONTRIBUTIONS

*Conceptualization*, Ling Shuai; *methodology*, Shengyi Sun, Qin Zhao, Yiding Zhao, Mengyang Geng, Qing Wang, Qian Gao; *software*, Xiao‐Ou Zhang; *formal analysis and writing*—*original draft*, Wenhao Zhang, and Ling Shuai; *writing—review & editing*, Ling Shuai; *supervision and funding acquisition*, Wenhao Zhang and Ling Shuai.

## CONFLICT OF INTEREST STATEMENT

The authors declare no conflict of interest.

## Supporting information


**Data S1:** Supporting Information

## Data Availability

The data that support the findings of this study are available from the corresponding author upon reasonable request. Raw data of RNA‐seq in this study have been deposited in the GenomeSequence Archive of the Beijing Institute of Genomics (BIG) Data Centre with project numbers PRJCA016665.
