# Supplementary material for: BCL2 is a major regulator of haploidy maintenance in murine embryonic stem cells
Source: Cell Prolif. 2023 May 5;56(12):e13498. doi: 10.1111/cpr.13498 (PMC10693186; doi:10.1111/cpr.13498)
Supplement: Supplementary file 1 — Data S1: Supporting Information [file CPR-56-e13498-s001.docx]

**Supplementary information**

**Title:** *BCL2* Is a Major Regulator of Haploidy Maintenance in Murine Embryonic Stem Cells

**Running title:** An Effective Method to Stabilize the Haploidy

**A****u****thors and affiliations:**

Shengyi Sun^1,5^, Qin Zhao^1,5^, Yiding Zhao^1^, Mengyang Geng^1^, Qing Wang^1^, Qian Gao^1^, Xiao-Ou Zhang^2,*^, Wenhao Zhang^1,3,*^ and Ling Shuai^1,4,*^.

1 State Key Laboratory of Medicinal Chemical Biology, College of Pharmacy, Tianjin Central Hospital of Gynecology Obstetrics/Tianjin Key Laboratory of Human Development and Reproductive Regulation, Nankai University, Tianjin 300350, China

2 School of Life Sciences and Technology, Tongji University, Shanghai 200092, China

3 Chongqing Key Laboratory of Human Embryo Engineering, Chongqing Health Center for Women and Children, Chongqing 400013, China

4 National Clinical Research Center for Obstetrics and Gynecology, Peking University Third Hospital, Beijing 100191, China

5 Co-first author

*** Correspondences:** zhangxiaoou@tongji.edu.cn; whzhang@nankai.edu.cn; lshuai@nankai.edu.cn (L.S.)

**Lead Contact:** lshuai@nankai.edu.cn (L.S.)


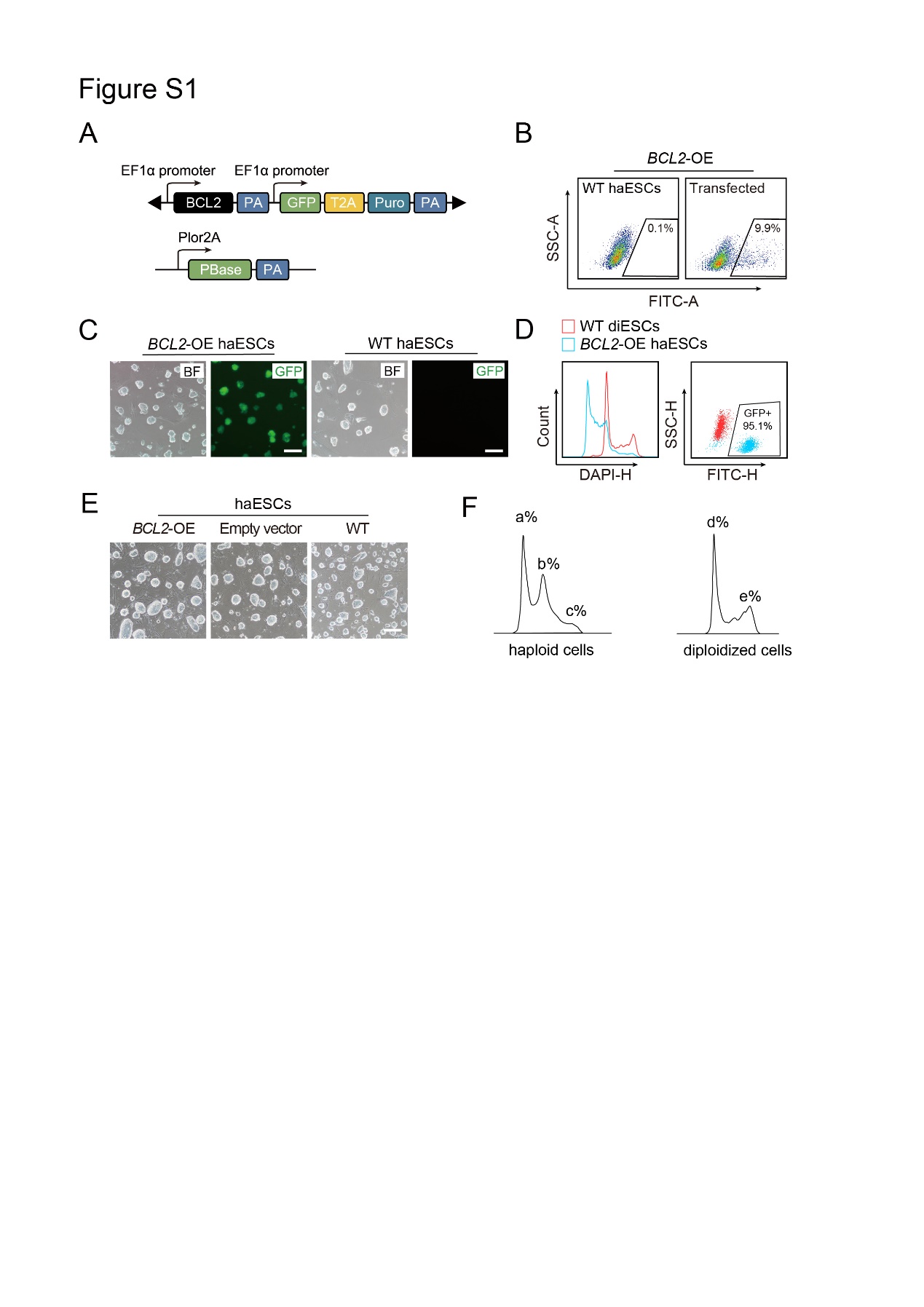


**Figure S1. OE of *BCL2* in HaESCs, Related to Figure 1.**

1. Schematic overview of the vectors used for *BCL2* OE.
2. Percentage of GFP positive cells in the electroporated haESCs 2 days after transfection as FACS indicated. WT haESCs without transfection are used as a negative control.
3. The bright field and GFP images of WT haESCs and *BCL2*-OE haESCs. Scale bar, 100 μm.
4. The haploid and GFP double positive of *BCL2*-OE haESCs as indicated by FACS. WT diploid ESCs (diESCs, GFP negative) are used as a control.

(E) Bright field images of *BCL2*-OE haESCs, empty vector transfected haESCs and WT haESCs. Scale bar, 100 μm.

(F) Cell cycle profiles of haploid cells and diploidized cells. The 1n-peak in haploid cells is the haploid cells with a 1-copy DNA set at G1 phase (a%); the 2n-peak in haploid cells is the haploid cells with a 2-copy DNA set at S-G2/M phase, mixing with diploid cells at G1 phase (b%); the 4n-peak in haploid cells is the diploid cells with a 2-copy DNA set at S-G2/M phase (c%); the 2n-peak in diploid cells is the diploid cells with a 2-copy DNA set at G1 phase (d%); the 4n-peak in haploid cells is the diploid cells with a 2-copy DNA set at S-G2/M phase (e%).


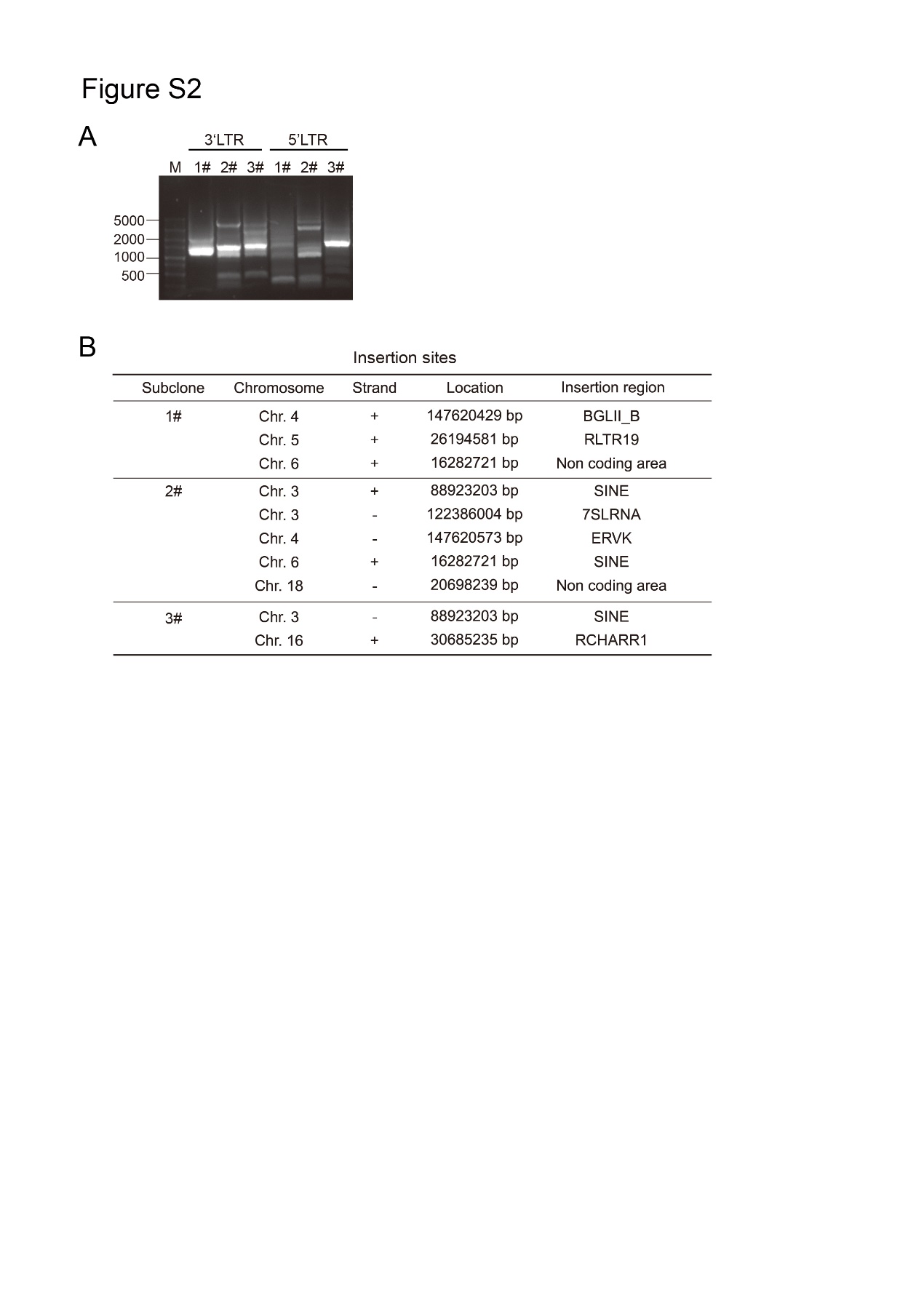


**Figure S2. Analysis of Insertion Sites of PB, Related to Figure 2.**

1. The inverse PCR products of PB 3’ LTR and 5’ LTR in *BCL2*-OE haESCs subclones 1#, 2# and 3#.
2. The results of Sanger sequencing indicate that all the checked insertion sites are in the inactive regions of the genome.


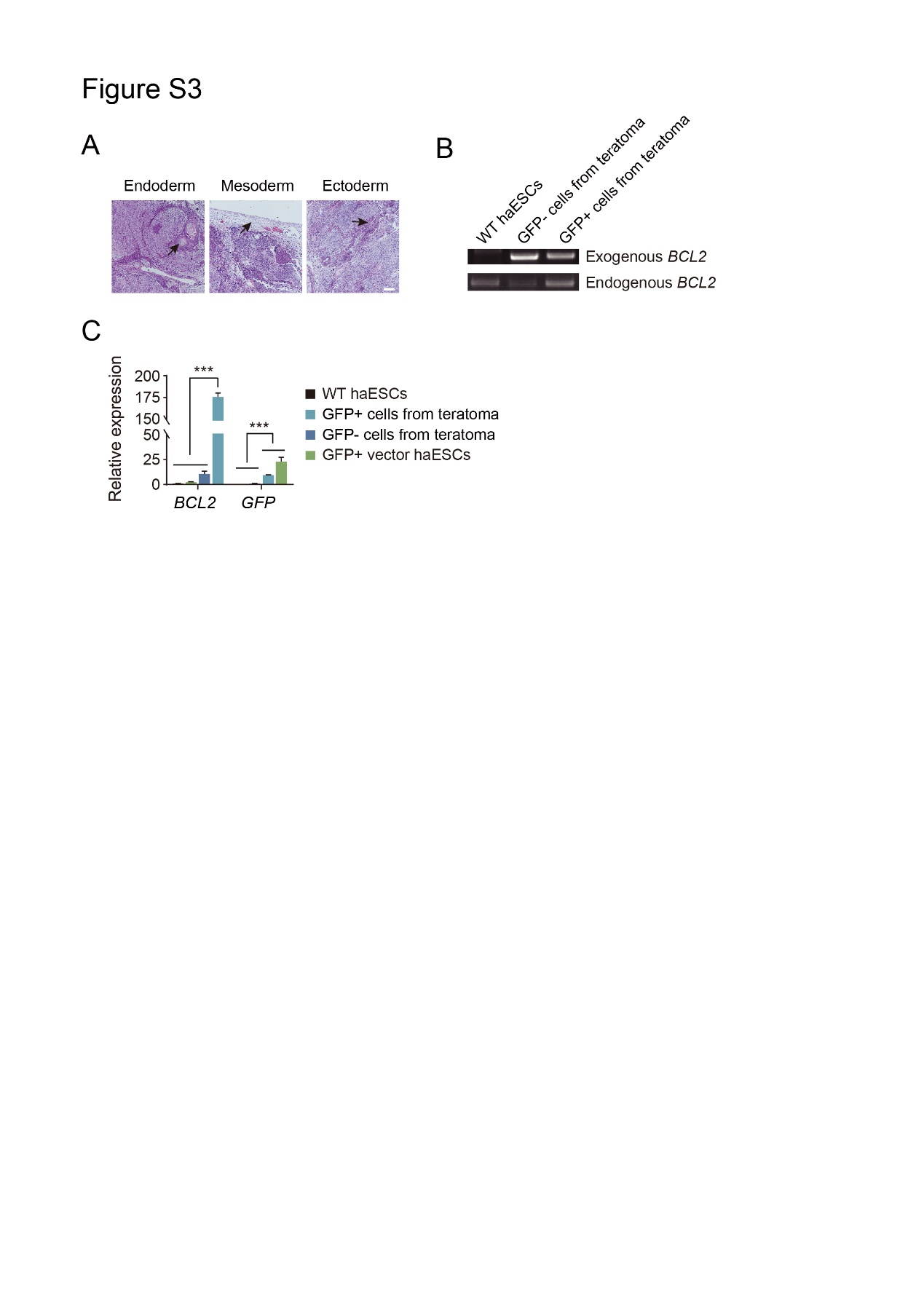


**Figure S3. Differentiation of *BCL2*-OE haESCs *In Vivo*, Related to Figure 3.**

1. H&E staining analysis of the teratoma formed from *BCL2*-OE haESCs. Scale bar, 100 μm. The tissues shown are gut glands, muscles and neural tubes.
2. The genotyping results of WT haESCs, GFP+ cells and GFP- cells from the *BCL2*-OE teratoma. Although *GFP* does not express in GFP- cells, exogenous *BCL2* is still detected in them.
3. The results of qPCR show that exogenous *BCL2* and *GFP* do not express in GFP- cells from the *BCL2*-OE teratoma. T test, **p* < 0.05, ***p* < 0.01, ****p* < 0.001. Data are represented as the mean ± SEM.


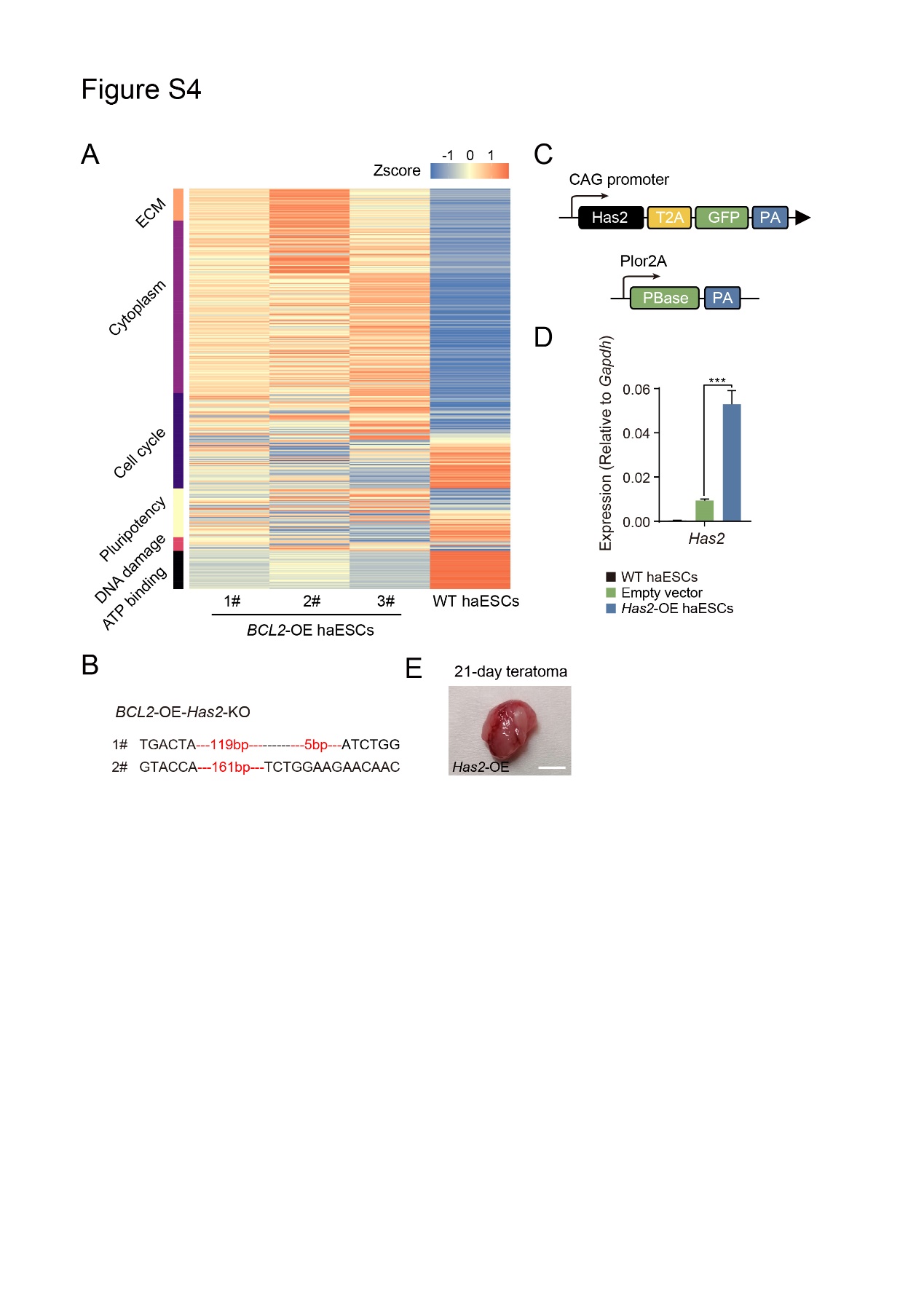


**Figure S4. Roles of *Has2* in *BCL2*-OE HaESCs and WT HaESCs, Related to Figure 5.**

1. The heatmap show that the expression levels in *BCL2*-OE haESCs are significantly different from those in WT haESCs in extracellular matrix and cytoplasm genes, while there are no significant changes in pluripotency, DNA damage and ATP binding genes.
2. The sequencing genotypes of *Has2* KO in the *BCL2*-OE haESCs.
3. Schematic overview of the vectors used for *Has2* OE.
4. The expression level of *Has2* in WT haESCs and *Has2*-OE haESCs. An empty *PB* vector electroporated haESCs is used as a control.
5. Teratoma formed from *Has2*-OE haESCs on Day 21. Scale bar, 1 cm.


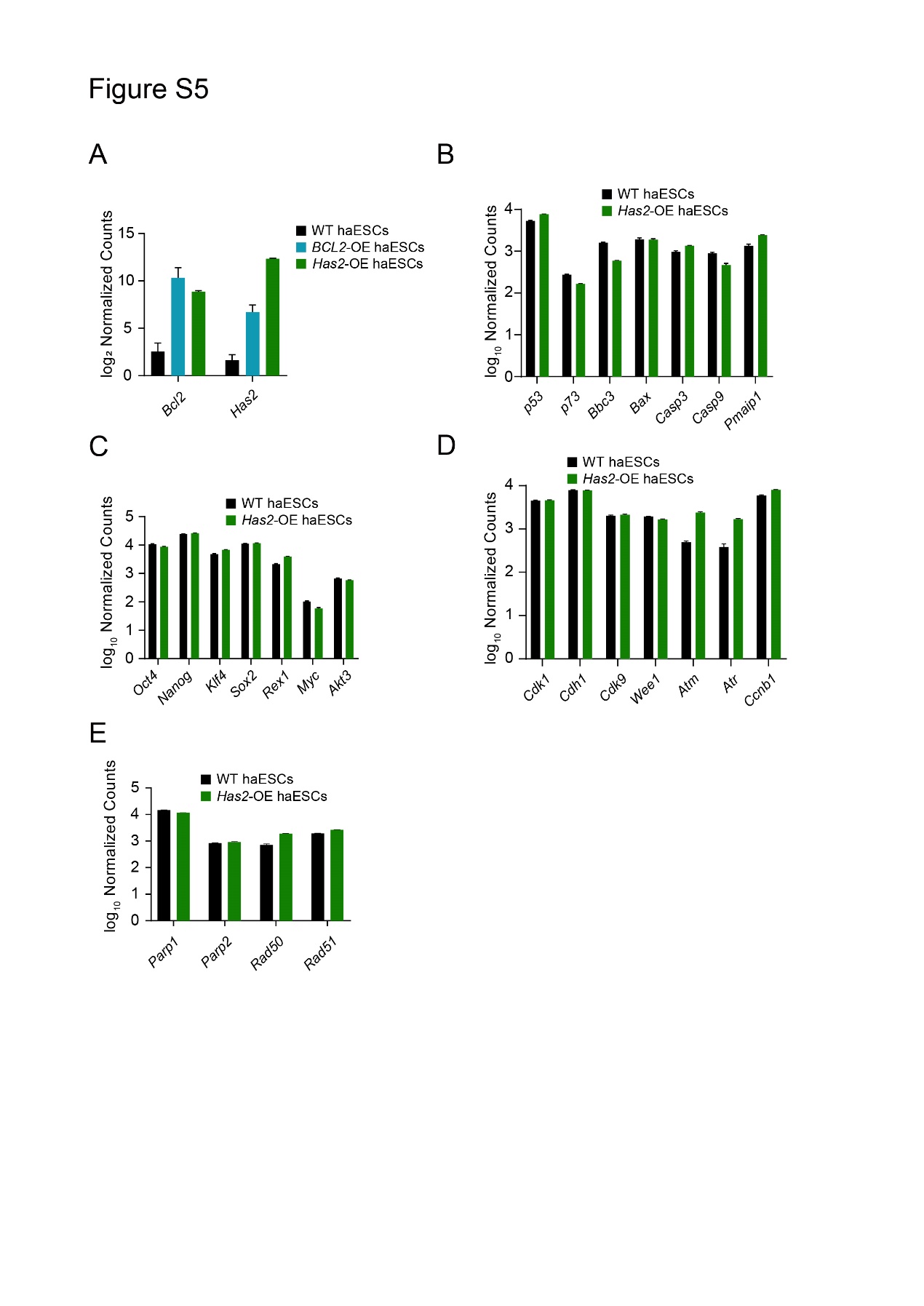


**Figure S5. Analysis of *Has2*-OE and *BCL2*-OE haESCs in Transcriptomes, Related to Figure 6.**

(A) The normalized count reads of *BCL2* and *Has2* in WT haESCs, *BCL2*-OE haESCs and *Has2*-OE haESCs.

(B) The normalized count reads of apoptosis related genes in WT haESCs and *Has2*-OE haESCs.

(C) The normalized count reads of pluripotent genes in WT haESCs, and *Has2*-OE haESCs. It shows that there is no significant difference in the expression of pluripotent genes between them.

(D) The normalized count reads of cell cycle related genes in WT haESCs and *Has2*-OE haESCs.

(E) The normalized count reads of DNA damage related genes in WT haESCs and *Has2*-OE haESCs.


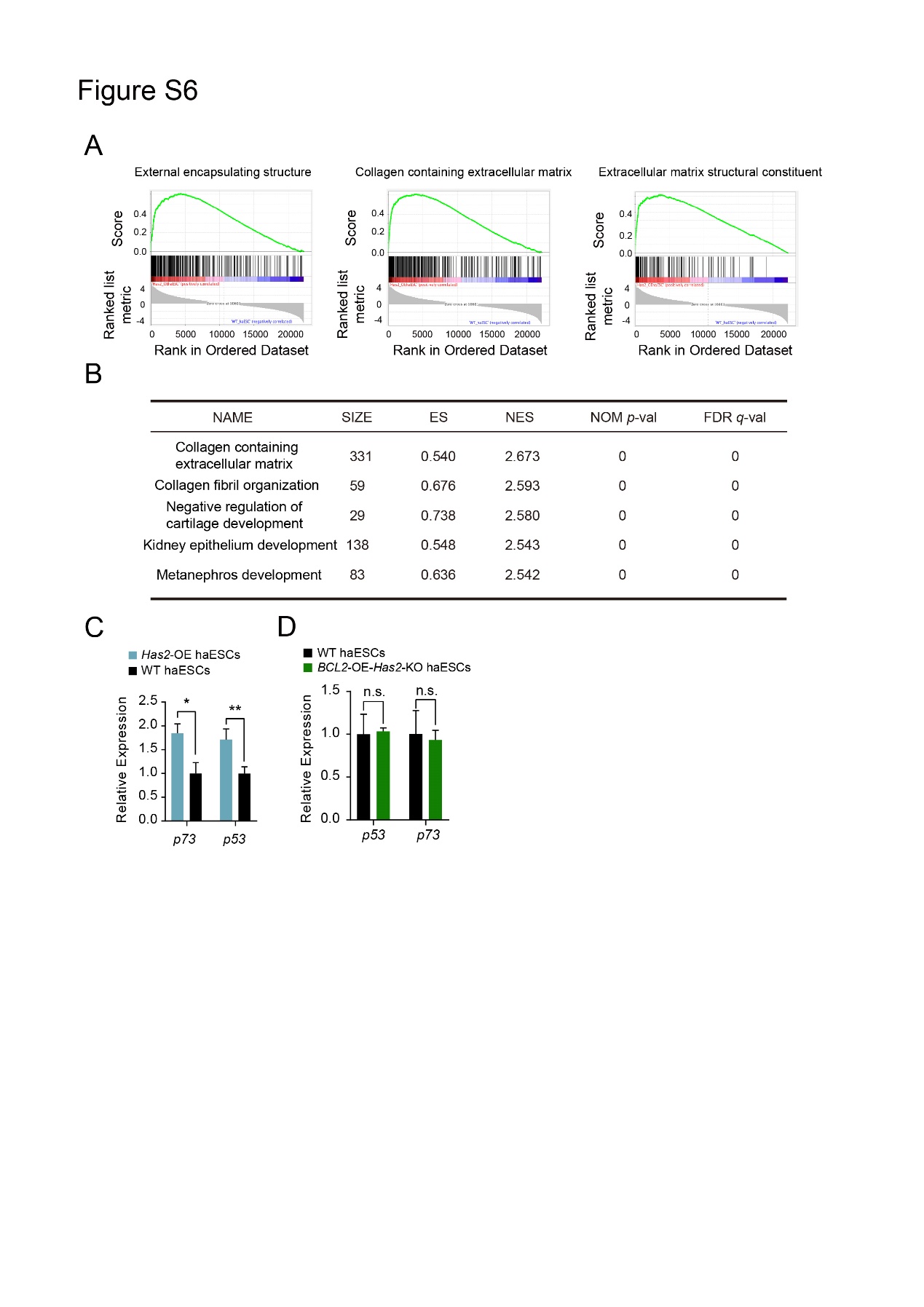


**Figure S6. Comparison of *BCL2*-OE and *Has2*-OE HaESCs, Related to Figure 6.**

(A) GSEA of all DEGs in *Has2*-OE haESCs is associated with External encapsulating structure, Collagen containing extracellular matrix and Extracellular matrix structural constituent.

(B) GSEA enrichment of DEGs in *BCL2*-OE haESCs.

(C) Expression levels of *p53* and *p73* in *BCL2*-OE haESCs and WT haESCs are determined by qPCR. T test, **p*< 0.05, ***p*< 0.01. Data are represented as the mean ± SEM.

(D) Expression levels of *p53* and *p73* in *BCL2*-OE-*Has2*-KO haESCs and WT haESCs determined by qPCR. T test, n.s., no significant difference. Data are represented as the mean ± SEM.

**Table S1. Primer Sequences**

| *BCL2* OE |  |  |
| --- | --- | --- |
|  | *BCL2*-Mlu I F | NNNNACGCGTATGGCGCAAGCCGGG |
|  | *BCL2*-Xba I R | NNNNTCTAGATCACTTGTGGCCCAGGTATGC |
| *Has2* OE |  |  |
|  | *Has2*-BamH I F | NNNNGGATCCATGCATTGTGAGAGGTTTCTATGTGTCC |
|  | *Has2*-BamH I R | NNNNGGATCCTCATACATCAAGCACCATGTCATACTGTTG |
| qPCR |  |  |
|  | *GAPDH*-F | AGGTCGGTGTGAACGGATTTG |
|  | *GAPDH*-R | TGTAGACCATGTAGTTGAGGTCA |
|  | *BCL2*-F | GGTGGTGGAGGAACTCTTCAGG |
|  | *BCL2*-R | GGAGAAATCAAACAGAGGTCGCATG |
|  | *Oct4*-F | TGCAGCTCAGCCTTAAGAACATG |
|  | *Oct4*-R | CCACCTCACACGGTTCTCAATG |
|  | *Nanog*-F | CAGCCTCCAGCAGATGCAAG |
|  | *Nanog*-R | TGCTGGGATACTCCACTGGTG |
|  | *Rex1*-F | CCCTCGACAGACTGACCCTAA |
|  | *Rex1*-R | TCGGGGCTAATCTCACTTTCAT |
|  | *Cyclin B*-F | GCACTTCCTCCGTAGAGCATC |
|  | *Cyclin B*-R | CATTCTTAGCCAGGTGCTGCATAAC |
|  | *Cdk2*-F | CCCAGAACCTGCTTATCAATGCAG |
|  | *Cdk2*-R | CAGATATCCACGGCTGTGGAGTAG |
|  | *Cdk1*-F | ACTTACACCAAATCCTCCAGGGAATT |
|  | *Cdk1*-R | GGTCCCTATACTCCAGATGTCAACC |
|  | *MAPK11*-F | GCGGGATTCTACCGGCAAG |
|  | *MAPK11*-R | GAGCAGACTGAGCCGTAGG |
|  | *Parp*-F | ACATCTCAGGGAGACCCGATTG |
|  | *Parp*-R | AACCTCTACACCCTCCAGGG |
|  | *Chk1*-F | CAATGCCTGAAAGAGACCTTCGAG |
|  | *Chk1*-R | CTGCTCACAACATCGCTGAGC |
|  | *Chk2*-F | ACCACAGAGGAGGCCTTAAATCATC |
|  | *Chk2*-R | CATTCAAAGCTCACAACACAGCC |
|  | *Fgf5*-F | ATCGGTTTCCATCTGCAGATCTACC |
|  | *Fgf5*-R | TCTCTTGTTCAGGGCCACGTAC |
|  | *Nodal*-F | CCTGTGGGGGAGGAGTTTCATC |
|  | *Nodal*-R | CCTGCCATTGTCCACATAAAGCATG |
|  | *Pax6*-F | AGCTTCACCATGGCAAACAACC |
|  | *Pax6-*R | CTGACTGTTCATGTGTGTTTGCATGTG |
|  | *Nestin-*F | TCGCTTGCAGACACCTGGAAG |
|  | *Nestin*-R | GTCACAGGAGTCTCAAGGGTATTAGG |
|  | *Cdx2*-F | GTCCCTAGGAAGCCAAGTGAA |
|  | *Cdx2*-R | TTGGCTCTGCGGTTCTGAAA |
|  | *Tfap2c*-F | ATCCCTCACCTCTCCTCTCC |
|  | *Tfap2c*-R | CCAGATGCGAGTAATGGTCGG |
|  | *P53*-F | CATGAACCGCCGACCTATCC |
|  | *P53*-R | GCAGTTCAGGGCAAAGGACT |
|  | *P73*-F | GGTTGGGGTGTCCAAACTGC |
|  | *P73*-R | CCTCCAGATGGTCATACGGTACTG |
|  | *Phlda3*-F | CCGTGGAGTGCGTAGAGAG |
|  | *Phlda3*-R | TCTGGATGGCCTGTTGATTCT |
|  | *CD44-F* | CCGCACTGTGACTCATGGATCC |
|  | *CD44-R* | CCCATTGCCACCGTTGATCAC |
|  | *Akt2*-F | GCTGGACTGCTGAAGAAGGAC |
|  | *Akt2*-R | CCTTGTGTCCACTTCTGAAGTGAC |
|  | *Has2*-F | GTTCACAACATGTCACCCAATTGG |
|  | *Has2*-R | CCTTCACCATCTCCACAGATGAGG |
|  | *GFP*-F | AGGAGCTGCACAGCAACAC |
|  | *GFP*-R | TGGGCTTGTACTCGGTCATAGG |
|  | miR-26b-3p-F | ttcaagtaattcaggataggt |
| Genotype |  |  |
|  | Endo *BCL2*-F | CGTCGCTACCGTCGTGAC |
|  | Endo *BCL2*-R | CACATGACCCCACCGAACTC |
|  | Exo *BCL2*-F | CGTCGCTACCGTCGTGAC |
|  | Exo *BCL2*-R | TAGGAAAGGACAGTGGGAGTGG |
|  | *Has2*-F | CTTTGCCTTTTTGGAACACCGG |
|  | *Has2*-R | CCTACCTGTACATAATCCACGCTTCG |
| inverse PCR |  |  |
|  | 1st 3’ PB-F | CCTCGATATACAGACCGATAAAACACATG |
|  | 1st 3’ PB-R | TCTTCTATAAAGTAACAAAACTTTTATGAGGGACAGC |
|  | 1st 5’ PB-F | GGTCATAGGGCCGGGATTC |
|  | 1st 5’ PB-R | GACTGAGATGTCCTAAATGCACAGC |
|  | 2nd 3’ PB-F | CGCATGATTATCTTTAACGTACGTCAC |
|  | 2nd 3’ PB-R | GGATACGGGGAAAAGGCCTC |
|  | 2nd 5’ PB-F | TCTCCTCCACGTCACCGC |
|  | 2nd 5’ PB-R | GAGCAATATTTCAAGAATGCATGCGTC |
| sgRNA |  |  |
|  | *Has2* sgRNA-1 | ACAAATCGGCCACGTACATC |
|  | *Has2* sgRNA-2 | CTGCATCGCTGCGTACCAAG |
|  | *Has2* sgRNA-3 | GGACATATTCAGCGAAGTTA |
|  | *Has2* sgRNA-4 | AAACATTTCCGTAAGTAGTC |
|  | *p53 sgRNA-1* | TAAGCCCTCAACACCGCCTG |
|  | *p53 sgRNA-2* | AAATTTGTATCCCGAGTATC |
|  | *p53 sgRNA-3* | CGAGGGCGTCCAATGGTGCT |
|  | *P53 sgRNA-4* | GCCACTGCGAGGGCGTCCAA |
